# Homeobox proteins are essential for fungal differentiation and secondary metabolism in *Aspergillus nidulans*

**DOI:** 10.1038/s41598-020-63300-4

**Published:** 2020-04-08

**Authors:** Sung-Hun Son, Ye-Eun Son, He-Jin Cho, Wanping Chen, Mi-Kyung Lee, Lee-Han Kim, Dong-Min Han, Hee-Soo Park

**Affiliations:** 1 School of Food Science and Biotechnology, Kyungpook National University, Daegu, 41566 Republic of Korea; 2 Department of Molecular Microbiology and Genetics, University of Göttingen, Göttingen, 37077 Germany; 3 Biological Resource Center (BRC), Korea Research Institute of Bioscience and Biotechnology (KRIBB), Jeongeup-si, 34141 Republic of Korea; 4 Division of Biological Sciences, Wonkwang University, Iksan, 54538 Republic of Korea; 5 Department of Integrative Biology, Kyungpook National University, Daegu, 41566 Republic of Korea

**Keywords:** Fungi, Microbial genetics

## Abstract

The homeobox domain-containing transcription factors play an important role in the growth, development, and secondary metabolism in fungi and other eukaryotes. In this study, we characterized the roles of the genes coding for homeobox-type proteins in the model organism *Aspergillus nidulans*. To examine their roles in *A. nidulans*, the deletion mutant strains for each gene coding for homeobox-type protein were generated, and their phenotypes were examined. Phenotypic analyses revealed that two homeobox proteins, HbxA and HbxB, were required for conidia production. Deletion of *hbxA* caused abnormal conidiophore production, decreased the number of conidia in both light and dark conditions, and decreased the size of cleistothecia structures. Overexpressing *hbxA* enhanced the production of asexual spores and formation of conidiophore under the liquid submerged conditions. The *hbxB* deletion mutant strains exhibited decreased asexual spore production but increased cleistothecia production. The absence of *hbxB* decreased the trehalose content in asexual spores and increased their sensitivity against thermal and oxidative stresses. The Δ*hbxA* strains produced more sterigmatocystin, which was decreased in the Δ*hbxB* strain. Overall, our results show that HbxA and HbxB play crucial roles in the differentiation and secondary metabolism of the fungus *A. nidulans*.

## Introduction

*Aspergillus nidulans* is a model filamentous fungus commonly used for the understanding of the genetics and molecular biology of fungal development and secondary metabolism^1–3^. The reproductive cycle of *A. nidulans* can be divided into two phases as asexual and sexual^4,5^. This fungus forms specialized developmental structures, which were used to designate the name of this fungus. Under the conditions that induce asexual reproduction, *A. nidulans* forms typical aspergillum-like structures called conidiophores, which contain a vesicle each, metulae, phialides, and conidia^6–8^. During the sexual cycle, *A. nidulans* (anamorph: *Emericella nidulans*) produces the nest-like sexual fruiting body called cleistothecium^9,10^. The formation of asexual or sexual structures involves sophisticated molecular events that are regulated by a variety of elements, especially transcription factors^4,5^.

Transcription factors have sequence-specific DNA-binding motifs and control the transcription levels of target genes^11^. To date, approximately 80 transcription factor families have been described in the fungal genome^12^. These transcription factors positively or negatively control gene expression during fungal development^5^. Several developmental activators (e.g., FlbB, FlbC, FlbD, and FlbE) or repressors (e.g., SfgA and NsdD) are involved in the activation of BrlA, an essential transcription factor initiating conidia formation and the asexual cycle^6,13,14^. BrlA contains a C_2_H_2_ zinc finger DNA-binding domain and controls the transcription of *abaA*, a key gene involved in the mid phase of conidiogenesis^15–17^. AbaA mainly governs the differentiation of phialides and activates the spore-specific transcription factor WetA^18–20^. WetA regulates the spore-specific gene expression during conidial formation and maturation, thereby controlling spore wall integrity and conidial maturation^21,22^. Alongside WetA, the two velvet transcription factors VosA and VelB are considered spore-specific transcription factors in *Aspergillus* species^23–26^. Various regulators have been reported to be involved in the sexual developmental processes as well^9^. For example, the Velvet protein complex (VeA-LaeA-VelB) orchestrates the formation of the sexual fruiting bodies and sterigmatocystin (ST) production^27,28^. Several transcription factors, including NosA, NsdC and NsdD, are also involved in sexual fruiting body formation and act as the activators of the sexual developmental cycle^29–31^. Although the functions of some transcription factors on fungal developmental processes have been reported, the roles of many other transcription factors are still unknown.

Homeobox proteins are highly conserved transcription factors that act as master developmental regulators in most multicellular organisms^32–34^. These proteins contain a 60–amino-acid–long homeodomain that contains the DNA-binding helix-turn-helix motif^35^, whereby they bind to their target genes involved in fungal differentiation^36^. By regulating the expression of such target genes, homeobox proteins govern differentiation in fungi^37^. Additionally, fungal homeobox proteins play important roles in fungal reproduction, secondary metabolism, and virulence^37^. Most Ascomycota or Basidiomycota fungi contain approximately 6–12 genes coding for homeobox-type proteins, which play diverse roles in fungal biology^37–39^. In the rice blast fungus *Magnaporthe oryzae*, eight genes coding for homeobox-type proteins have been characterized. Several of them, such as *MoHOX2* and *MoHOX7*, are required for asexual reproduction, appressorium development, and pathogenesis^40,41^. In *Saccharomyces cerevisiae*, homeobox transcription factors are involved in filamentous growth, meiosis, and mating^37,42^. *GRF10* has been shown to control the filamentous growth and pathogenicity of the human fungal pathogen *Candida albicans* in a mouse model^43^.

Recently, the roles of fungal homeobox transcription factors have been characterized in two *Aspergillus* species, *A. fumigatus* and *A. flavus*^44–46^. In the aflatoxin-producing fungus *A. flavus*, eight genes coding for homeobox-type proteins have been characterized. Among them, Hbx1 is required for fungal development and the production of aflatoxins^44^. Deletion of *hbx1* causes loss of conidiophore and sclerotium formation and aflatoxin production^44^. Transcriptomic analysis has demonstrated that Hbx1 regulates the expression of developmental regulatory genes and secondary metabolite gene clusters^45^. In *A. fumigatus*, the Hbx1 homolog HbxA is also involved in the asexual development, production of several secondary metabolites, and virulence^46^. Overall, the roles of homeobox transcription factors have been characterized only in these two *Aspergillus* species but not in the model fungus *A. nidulans* yet. Here, we identified genes coding for homeobox-type proteins in *A. nidulans* and characterized their roles via the phenotypic analyses of deletion mutants. Among them, the two homeobox transcription factors HbxA and HbxB were further functionally characterized. We found that these two proteins were required for the reproduction and ST metabolism of *A. nidulans*. In addition, HbxB governs the trehalose biosynthesis and stress tolerance in *A. nidulans* conidia.

## Results

### Genes coding for homeobox-type proteins in *A. nidulans*

**Figure 1:**
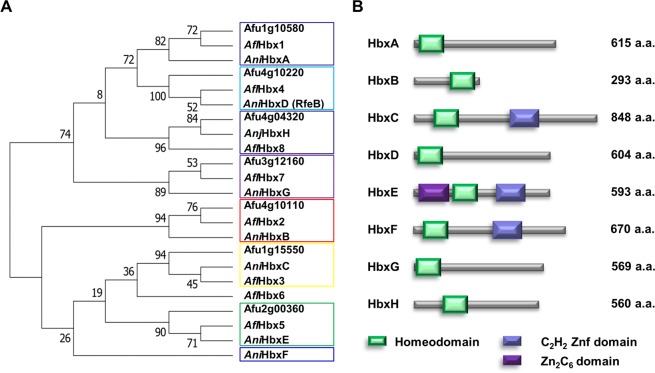
The phylogenetic analyses and sequence features of the Homeobox proteins in *A. nidulans*. **(A)** The phylogenetic tree of the putative homeobox proteins in three *Aspergillus* species, including *A. nidulans* FGSC4, *A. fumigatus* AF293, and *A. flavus* NRRL 3357. **(B)** The domain architecture of the putative homeobox proteins in *A. nidulans*.

To identify genes coding for homeobox-type proteins in *A. nidulans* genome, the protein sequence of the homeobox domain (IPR001356) was queried into the ASPGD database (www.aspgd.org). Consequently, eight genes were identified in the genome of *A. nidulans* FGSC4. To name these genes, their predicted protein sequences were aligned with the sequences of *A. fumigatus* and *A. flavus* homeobox proteins using Clustal Omega (https://www.ebi.ac.uk/Tools/msa/clustalo/), and the aligned sequences were input to MEGA software. Based on *A. flavus* homeobox proteins, the names of HbxA–HbxF were chosen **(**Fig. 1A**)**. Domain analysis found that these proteins contained a 60–amino-acid–long helix-turn-helix DNA-binding domain **(**Fig. S1**)**. Three proteins, HbxC, HbxE and HbxF, have a C_2_H_2_ zinc finger domain at their C termini. In addition, HbxE contains a GAL4-like Zn(2) C_6_ fungal type DNA binding domain **(**Fig. 1B**)**.

### The roles of homeobox proteins in fungal development

**Figure 2:**
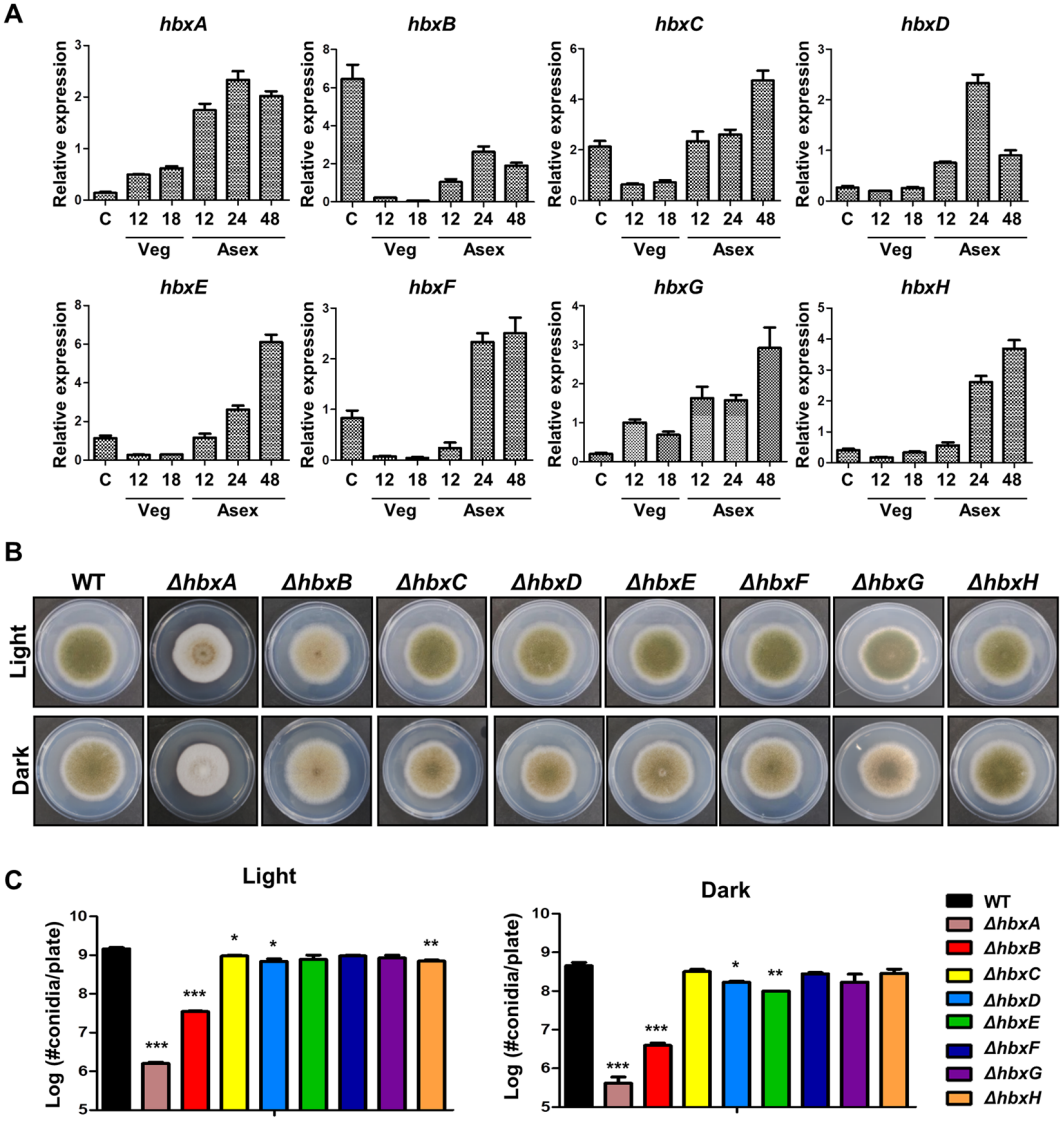
The roles of eight genes coding for homeobox-type proteins in *A. nidulans*. **(A)** The expression level of each gene coding for homeobox-type protein during the life cycle of *A. nidulans* was measured by qRT-PCR. C = Conidia, Veg = Vegetative growth, Asex = Asexual development. **(B)** The colony photographs of WT or deletion mutant strains point-inoculated on solid MMG and grown for 5 days at 37 °C in the light or dark. **(C)** Quantitative analysis of the conidia in the WT and mutant strains after 5 days of incubation at 37 °C in the light or dark. Differences between the WT and mutants, *p < 0.05, **p < 0.01, and ***p < 0.001).

To predict the functions of *hbxA*–*hbxF*, mRNA levels of these genes were examined during the life cycle of *A. nidulans*. As shown in Fig. 2A, *hbxA*–*hbxF* were expressed during asexual development. Especially, *hbxB* mRNA levels were high in conidia. To further study the roles of genes coding for homeobox-type proteins, deletion mutants for each *hbx* gene were generated, and their developmental phenotypes were examined. WT or mutant strains were point-inoculated onto MMG or SM and cultured in the light or dark **(**Fig. 2B**)**. In both conditions, the colony phenotypes of the Δ*hbxA* and Δ*hbxB* mutant strains were different from those of the WT strains. Importantly, the Δ*hbxA*, Δ*hbxB*, and Δ*hbxD* strains produced significantly less amount of conidia than the WT strains under both light and dark conditions **(**Fig. 2C**)**. Taken together, *hbxA* and *hbxB* seem to play important roles in fungal development, and thus the functions of *hbxA* and *hbxB* were further studied.

### The roles of *hbx*A in development

**Figure 3:**
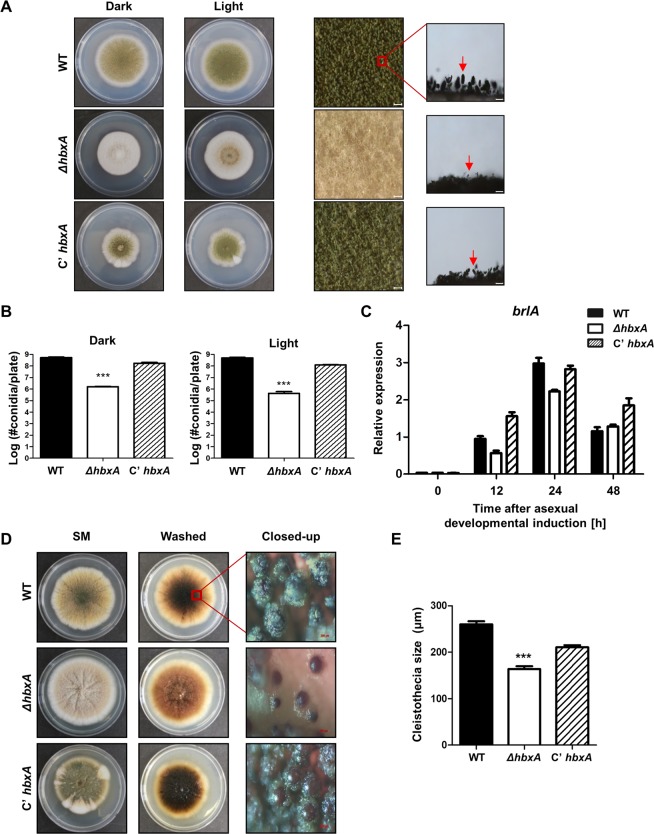
Fungal development of the *ΔhbxA* mutant. **(A)** Colony morphology of WT (TNJ36), *ΔhbxA* (TYE14), and *C’hbxA* (TYE27) strains grown on MMG for 5 days at 37 °C in the light or dark. The middle panels show the magnified views of the middles of the plates (bar = 0.25 µm). The panels on the right show the morphologies of the WT and mutant conidiophores under a microscope (bar = 0.25 µm). Arrows indicate conidiophores. **(B)** Quantitative analysis of the conidia shown in (A). (Differences between the WT and mutants, ***p < 0.001). **(C)** qRT-PCR analysis for *brlA* mRNA levels in WT (TNJ36), *ΔhbxA* (TYE14), and *C’hbxA* (TYE27) strains after inducing asexual development. β-Actin was used as the endogenous control. **(D)** WT (TNJ36), *ΔhbxA* (TYE14), and *C’hbxA* (TYE27) strains were point-inoculated, and the plates were grown on SM for 7 days at 37 °C in the dark. The middle panels show the plates, which were washed with ethanol to observe the sexual structure. The panels on the right show the magnified views of the sexual structures in WT (TNJ36), *ΔhbxA* (TYE14), and *C’hbxA* (TYE27) strains (bar = 0.25 µm). **(E)** The sizes of the cleistothecia in WT (TNJ36), *ΔhbxA* (TYE14), and *C’hbxA* (TYE27) strains (differences between the WT and mutants, ***p < 0.001).

To further assess the roles of *hbxA*, *hbxA*-complemented strains (*C’hbxA*) were generated. WT, Δ*hbxA*, and *C’hbxA* strains were point-inoculated onto MMG and their conidiophore structures were examined. Under the light condition, the Δ*hbxA* strain exhibited abnormal conidiophore morphology **(**Fig. 3A**)**. The number of conidia in Δ*hbxA* strain was significantly less than in WT and *C’hbxA* strains **(**Fig. 3B**)**. To test whether the deletion of *hbxA* affected the expression pattern of *brlA*, a key gene for asexual development, the mRNA levels of *brlA* in WT, Δ*hbxA*, and *C’hbxA* strains grown under the conditions that preferentially induce asexual development were examined. As shown in Fig. 3C, the deletion of *hbxA* reduced *brlA* expression by 12 h and 24 h of the induction of asexual development.

To further investigate the roles of HbxA in sexual development, these strains were inoculated onto SM. WT and *C’hbxA* strains produced normal sexual fruiting bodies, whereas the Δ*hbxA* strains formed small and abnormal cleistothecia **(**Fig. 3D**)**. The size of cleistothecia in the Δ*hbxA* strain was smaller than that of WT or *C’hbxA* strains **(**Fig. 3E**)**. Taken together these results demonstrated that HbxA was essential for sexual development in *A. nidulans*.

### Overexpression of *hbxA* leads to enhanced conidiation

**Figure 4:**
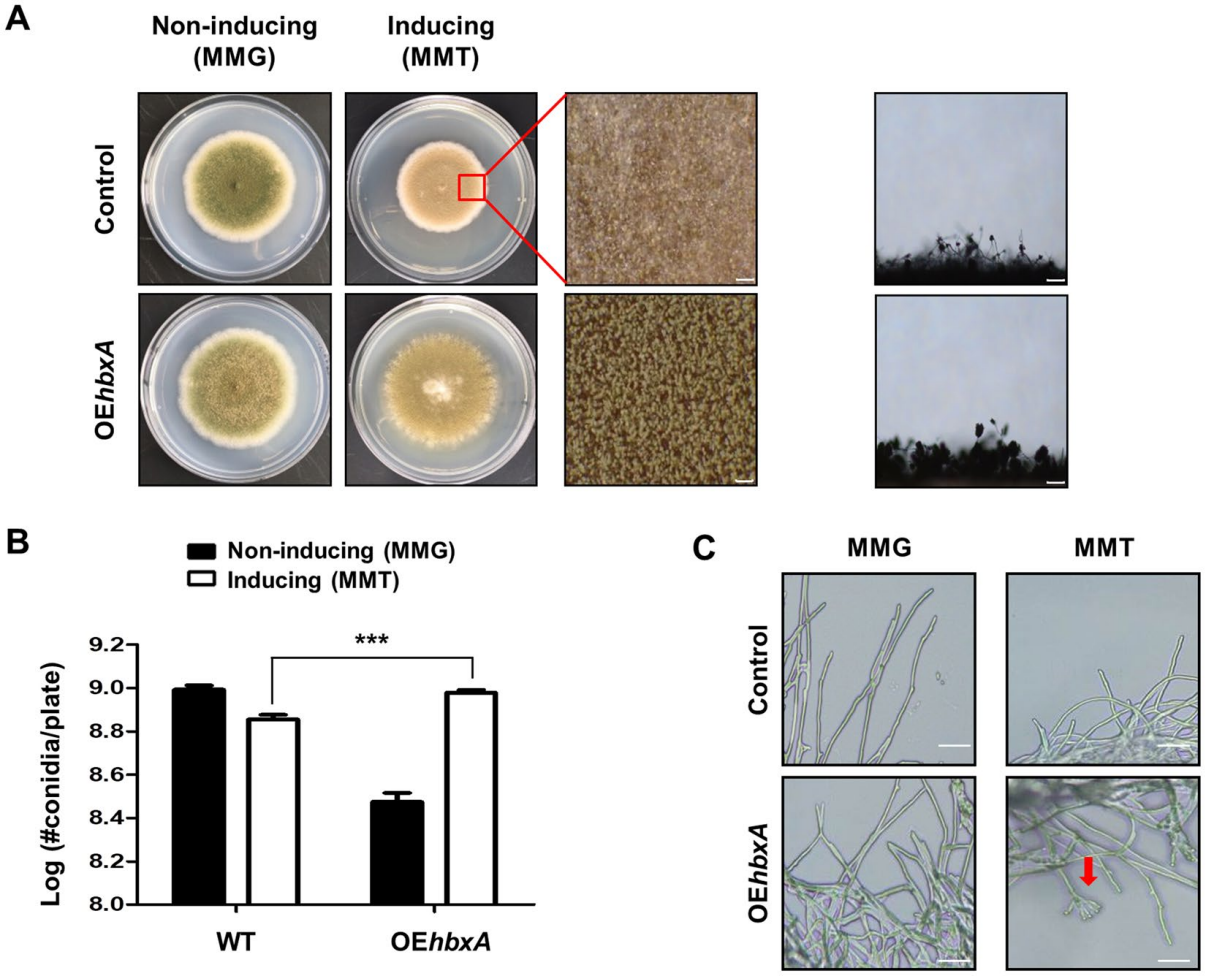
Effect of *hbxA* overexpression. **(A)** Control (TNJ36) and *hbxA*–overexpression (TYE19) strains were inoculated onto non-inducing (MMG) or inducing (MMT) condition media and photographed after 5 days of culture. The middle panels show the magnified views of the middles of the plates under the inducing conditions (bar = 0.25 µm). The panels on the right show the morphologies of the control (TNJ36) and OE*hbxA* (TYE19) conidiophores under the inducing conditions (bar = 0.25 µm). **(B)** Quantification of the number of conidia in the control (TNJ36) and OE*hbxA* (TYE19) strains shown in (A). Differences between the control and mutants, ***p < 0.001. **(C)** Photomicrographs of the mycelia in the control (TNJ36) and OE*hbxA* (TYE19) strains grown in liquid MMG (non-inducing) or MMT (inducing) media. Arrow indicates conidiospore.

Because the absence of *hbxA* affected fungal development, we then tested whether the overexpression of *hbxA* could influence fungal development. To test this hypothesis, *hbxA* overexpression (OE*hbxA*) mutant strains were constructed. WT and OE*hbxA* strains were point-inoculated onto non-inducing (MMG) or inducing media (MMT), and their asexual developmental phenotypes were examined **(**Fig. 4A**)**. Under the inducing conditions, the overexpression of *hbxA* enhanced the production of conidiospores **(**Fig. 4B**)**. To further confirm this observation, OE*hbxA* strains were inoculated into liquid MMT (inducing condition). Whereas WT strains could not develop conidiophores in either of the inducing or non-inducing conditions, the OE*hbxA* strain exhibited the formation of conidiophores in liquid submerged culture **(**Fig. 4C**)**. These results demonstrated that HbxA could act as an activator of asexual development in *A. nidulans*.

### Deletion of *hbxB* enhances sexual development

**Figure 5:**
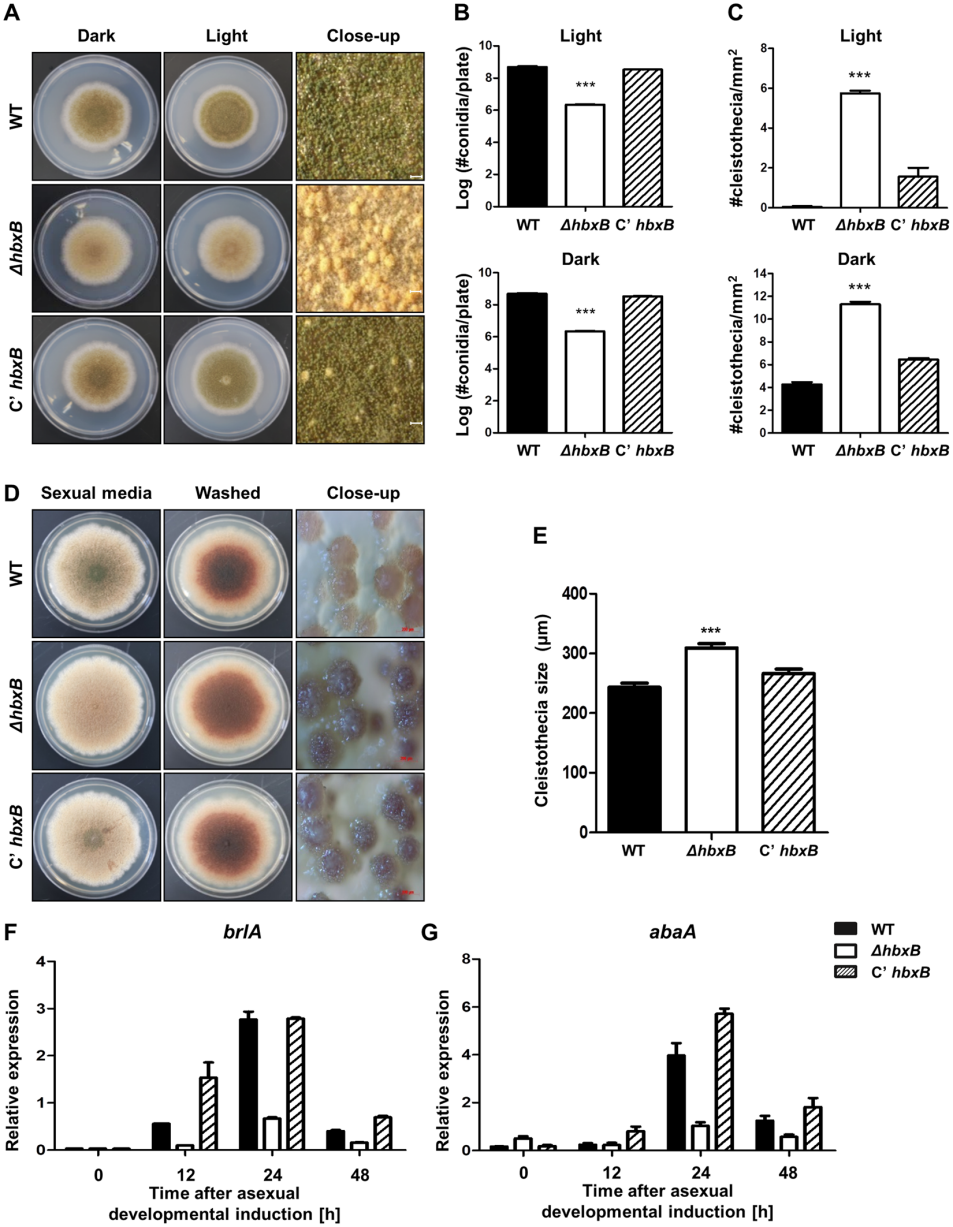
Developmental phenotypes of the *ΔhbxB* mutant. **(A)** The colony photographs of WT(TNJ36), *ΔhbxB* (TSH1), and *C’hbxB* (TSH7) strains point-inoculated on solid MMG and grown for 5 days at 37 °C in the light or dark. The panels on the right show the magnified views of the plates grown in the light (bar = 0.25 µm). **(B)** Quantitative analysis of the number of conidia from WT(TNJ36), *ΔhbxB* (TSH1), and *C’hbxB* (TSH7) strains shown in (A). The number of conidia per plate was counted in triplicate. Differences between the WT and mutants, ***p < 0.001. **(C)** Quantitative analysis of the number of cleistothecia from WT (TNJ36), *ΔhbxB* (TSH1), and *C’hbxB* (TSH7) strains shown in (A). Differences between the WT and mutants, ***p < 0.001. **(D)** The colony morphologies of WT (TNJ36), *ΔhbxB* (TSH1), and *C’hbxB* (TSH7) strains after 7 days of culture at 37 °C in the dark. The colonies were washed to enable the visualization of the sexual structures (middle panels) and the magnified views of the edges of the plates (right panels, bar =200 μm). **(E)** The size of WT (TNJ36), *ΔhbxB* (TSH1), and *C’hbxB* (TSH7) strains. Differences between the WT and mutants, ***p < 0.001. **(F,G)** qRT-PCR analysis for *brlA* **(F)** and *abaA* **(G)** mRNA levels in WT (TNJ36), *ΔhbxB* (TSH1), and *C’hbxB* (TSH7) strains after inducing asexual development. β-Actin was used as the endogenous control.

As mentioned above, the deletion of *hbxB* decreased the number of conidia **(**Fig. 2**)**, suggesting that HbxB may act as a developmental regulator. To test this hypothesis, WT, Δ*hbxB*, and *hbxB*-complemented strains (*C’hbxB*) were inoculated onto MM plates and cultured in the light or dark **(**Fig. 5A**)**. Under both dark and light conditions, the Δ*hbxB* strains produced fewer conidia than WT or *C’hbxB* strains **(**Fig. 5B**)**. However, the number of cleistothecia in the Δ*hbxB* strains was increased compared with that of WT or *C’hbxB* strains **(**Fig. 5C**)**. The cleistothecia in the Δ*hbxB* strains were bigger than that of WT or *C’hbxB* strains under the conditions that induced sexual development **(**Fig. 5D,E**)**. To further elucidate the developmental role of HbxB, the mRNA levels of two key developmental activators, BrlA and AbaA, were examined during asexual developmental processes. As shown in Fig. 5F, *brlA* mRNA levels were decreased in Δ*hbxB* strains after inducing asexual development. The *abaA* mRNA levels in Δ*hbxB* strains were also decreased by 24 and 48 h of the asexual development induction. Overall, these results suggest that HbxB is required for a correct timing of development and the balance between both developmental programs.

### Overexpression of *hbxB* causes enhanced conidiation

**Figure 6:**
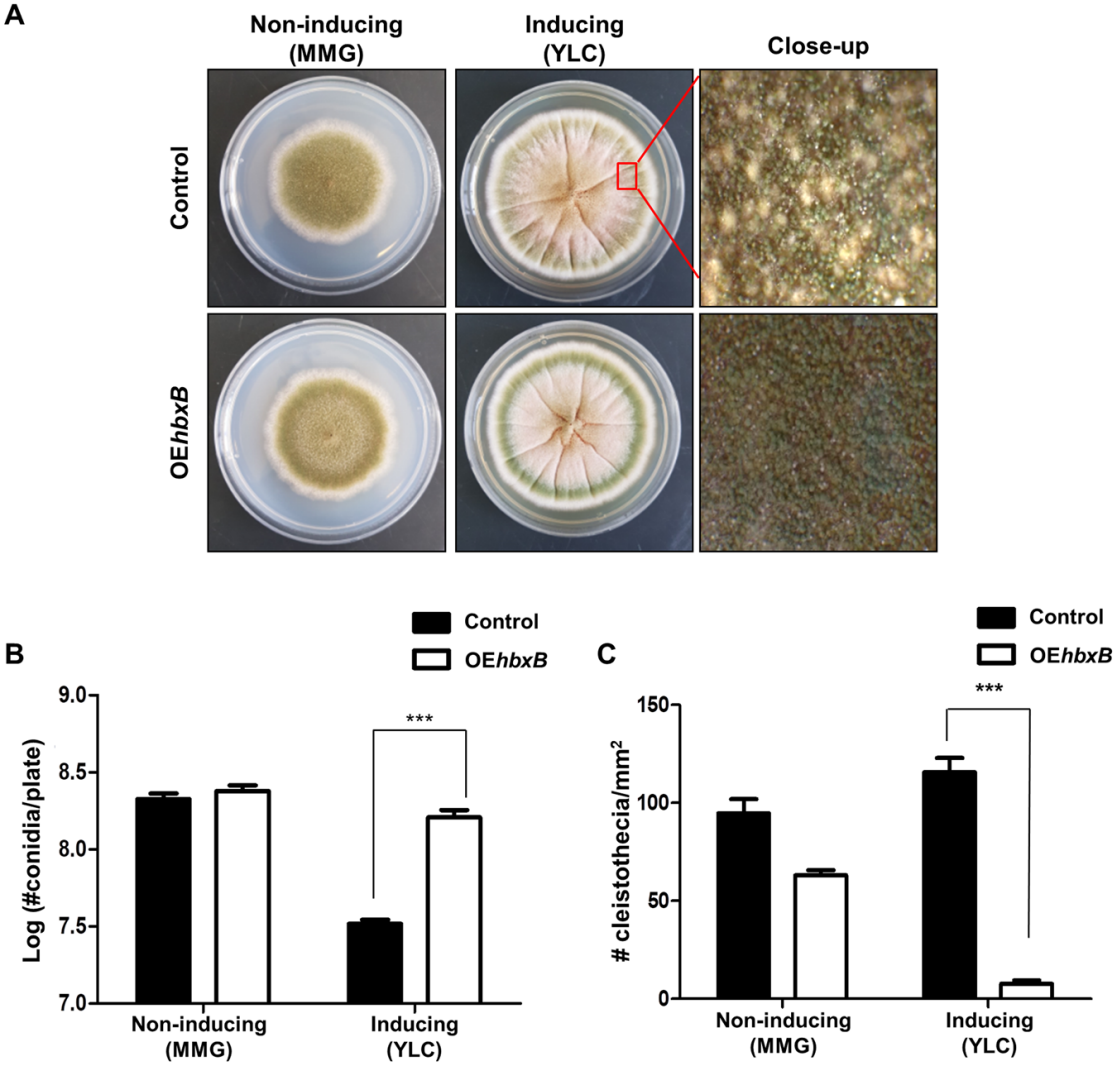
Developmental phenotypes of the *hbxB* overexpression strain. **(A)** Control (TNJ36) and *hbxB*–overexpression (TSH13) strains were inoculated onto non-inducing (MMG) or inducing (YLC) condition media and photographed after 5 days of culture. The right panels show the magnified views of the middles of the plates under the inducing conditions (bar = 0.25 µm). **(B–C)** Quantification of the number of conidia (**B**) or cleistothecia (**C**) in the control (TNJ36) and OE*hbxB* (TSH13) strains shown in (**A**). Differences between the control and mutants, ***p < 0.001. All the experiments were carried out in triplicate.

To further investigate the role of *hbxB* in development, *hbxB* overexpression (OE*hbxB*) mutant strains were generated and checked the production of asexual spores. Whereas control strains can produce lots of sexual fruiting bodies, OE*hbxA* strains exhibited less sexual fruiting bodies under the inducing condition **(**Fig. 6A**)**. In addition, the overexpression of *hbxB* increased the production of asexual spores **(**Fig. 6B**)**, but decreased the production of cleistothecia **(**Fig. 6C**)**. Taken together, these results propose that HbxB can act as a balancer between sexual and asexual development.

### The roles of *hbx*B in trehalose biosynthesis in conidia

**Figure 7:**
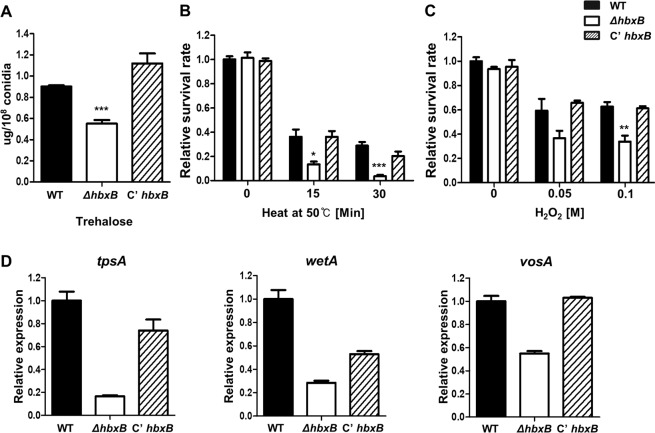
The role of *hbxB* in trehalose biosynthesis and stress tolerance. **(A)** Trehalose amount of conidia in WT (TNJ36), *ΔhbxB* (TSH1), and *C’hbxB* (TSH7) strains (measured in triplicate) (***p < 0.001). **(B)** Tolerance of WT (TNJ36), *ΔhbxB* (TSH1), and *C’hbxB* (TSH7) conidia to thermal stress (50 °C, triplicate measurements). Differences between the WT and mutants, *p < 0.05, ***p < 0.001. **(C)** The oxidative stress response of WT (TNJ36), *ΔhbxB* (TSH1), and *C’hbxB* (TSH7) conidia (triplicate measurements). Differences between the WT and mutants, **p < 0.01. **(D)** The mRNA levels of *tpsA*, *wetA*, and *vosA* in WT (TNJ36), *ΔhbxB* (TSH1), and *C’hbxB* (TSH7) conidia.

As mentioned above, *hbxB* mRNA levels were high in conidia **(**Fig. 2A**)**, implying that HbxB may participate in conidial formation and maturation. To test this hypothesis, trehalose content and stress tolerance were examined. The conidia of the Δ*hbxB* mutant strains contained less trehalose than the conidia of the WT or *C’hbxB* strains **(**Fig. 7A**)**. Because trehalose is a key protective factor against environmental stresses^47,48^, the conidial tolerance against thermo and oxidative stresses was tested. At high temperature or high concentration of H_2_O_2_, the viability of the Δ*hbxB* mutant conidia was decreased compared to WT or *C’hbxB* conidia **(**Fig. 7B,C**)**. We then examined the mRNA levels of *tpsA*, *wetA*, and *vosA*, which are associated with trehalose biosynthesis. As shown in Fig. 7D, the Δ*hbxB* mutant conidia exhibited decreased mRNA levels of *tpsA*, *wetA*, and *vosA* than WT or *C’hbxB* conidia. Overall, these results demonstrated that HbxB was a key regulator of trehalose biosynthesis.

### The roles of the *hbxA* and *hbxB* genes in ST production

**Figure 8:**
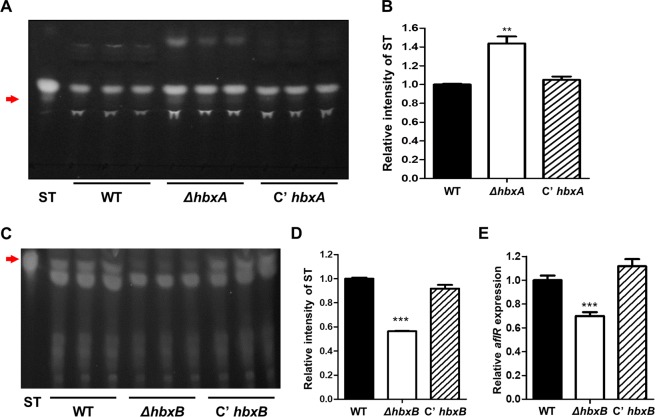
Analysis of ST production in the *ΔhbxA* and *ΔhbxB* mutant. **(A)** Thin-layer chromatography (TLC) of ST from WT (TNJ36), *ΔhbxA* (TYE14), and C’*hbxA* (TYE27) strains was performed for 7 days in the dark. Arrow indicates ST. **(B)** Densitometry of the ST bands from the TLC plates shown in (**A**). Differences between the WT and mutants, **p < 0.01. **(C)** Thin-layer chromatography (TLC) of ST from WT (TNJ36), *ΔhbxB* (TSH1), and *C’hbxB* (TSH7) strains was performed for 7 days in the dark. Arrow indicates ST. **(D)** The densitometry of the ST bands from the TLC plates shown in (**C)**. Differences between the WT and mutants, ***p < 0.001. **(E)** Relative mRNA levels of *aflR* in WT (TNJ36), Δ*hbxB* (TSH1), and C’*hbxB* (TSH7) strains.

Because the *hbxA* homolog *hbx1* is essential for aflatoxin production in *A. flavus*^44^, ST production in the absence of genes coding for homeobox-type proteins was examined. Interestingly, the deletion of *hbxA* or *hbxB* affected production of ST **(**Fig. S2**)**. Therefore, the roles of *hbxA* and *hbxB* in ST production were examined. ST in WT, Δ*hbxA*, and C’ *hbxA* were extracted and these samples were analyzed using TLC analysis. As shown in Fig. 8A,B, the Δ*hbxA* mutant strains produced more ST than WT or C’ *hbxA* strains, suggesting that HbxA may negatively affect ST production. To verify this observation, ST production was examined in WT, Δ*hbxB*, and *C’hbxB* strains. Indeed, the ST production in the Δ*hbxB* mutant was lower than in WT and *C’hbxB* strains **(**Fig. 8C,D**)**. In addition, the mRNA level of *aflR*, a transcriptional activator of the ST biosynthesis gene cluster, was also decreased **(**Fig. 8E**)**. Collectively, these results demonstrated that HbxB was necessary for proper ST biosynthesis.

## Discussion

Homeobox transcription factors are evolutionarily conserved proteins that play vital roles in the developmental processes of multicellular organisms, including Ascomycota and Basidiomycota^37^. These genes have been found to reside at the mating type loci of the fungal genomes and associate with fruiting body formation in various fungal species^37^. In addition, genes coding for homeobox-type proteins are associated with various fungal processes, including conidiation, virulence, and secondary metabolisms. Here, we characterized the functions of genes coding for homeobox-type proteins in the model fungus *A. nidulans*.

We identified eight genes coding for homeobox-type proteins in the *A. nidulans* genome. Among them, *hbxA* had the greatest effects on the development of *A. nidulans*. In the absence of *hbxA*, the conidiophore formation decreased along with decreased mRNA level of *brlA*, a key transcription factor for the initiation of conidiogenesis, during the early phase of conidiogenesis **(**Fig. 3**)**, implying that HbxA is necessary for the proper asexual development. Similar results have been reported in other *Aspergillus* species^40,46^. In *A. fumigatus*, the Δ*hbxA* mutant strains exhibit an almost aconidial phenotype with fewer conidia. Moreover, the *brlA* mRNA level is also decreased in this mutant strain^46^. Deleting the *hbxA* homolog *hbx1* shows similar results in *A. flavus*^44,45^. In other fungi, such as *M. oryzae*, *Fusarium graminearum*, and *Ustilaginoidea virens*, the putative *hbxA* orthologs (although similarity was low) are also involved in conidiogenesis. In *M. oryzae*, the deletion of *Mohox2* results in no conidia production^40^. The *Fghtf1* null mutant produces abnormal macroconidia in *F. graminearum*^49^. *UvHox2* deletion mutants of *U. virens* also produce abnormal asexual structures^38^. Overall, these results suggest that HbxA has conserved roles in asexual development in fungal species.

Although HbxA plays a conserved role in asexual development, its role in secondary metabolism differs among fungi. Our results revealed the Δ*hbxA* mutant strains produced ST, a known aflatoxin precursor in *A. nidulans* **(**Fig. 8**)**. However, aflatoxin and aflatrem production is abolished in the Δ*hbx1* mutant of *A. flavus*^44^. The Δ*hbxA* mutant of *A. fumigatus* produces less secondary metabolites, including fumigaclavines, fumiquinazolines, and chaetominine^46^. In both *A. flavus* and *A. fumigatus*, the *hbxA* homologs are involved in the regulation of secondary metabolism. Although we measured ST production in these mutants, we did not check the whole chemical profiles in the mutants. Therefore, additional research should be conducted to elucidate the mechanism(s) underlying the HbxA and HbxB control of secondary metabolite production.

Unlike other genes coding for homeobox-type proteins, *hbxB* was highly transcribed in conidia **(**Fig. 2**)**, suggesting that HbxB is involved in conidial maturation. As shown in Fig. 5, the Δ*hbxB* mutant conidia contained less trehalose and were more sensitive to thermal and oxidative stresses. In addition, the mRNA levels of the trehalose synthase gene (*tpsA*) and two spore-specific transcription factors (*wetA* and *vosA*) were decreased in the Δ*hbxB* mutant conidia. These observations suggest that HbxB can be a spore-specific gene. HbxB also affected fungal development. The deletion of *hbxB* increased the number of sexual fruiting bodies but decreased conidia number. Moreover, developmental phenotypes of OE*hbxB* strains exhibited the opposite phenotypes of Δ*hbxB* mutant strains, suggesting that HbxB acts as a balancer between the asexual and sexual developments in *A. nidulans*.

Although the functions of six (*hbxC*-*hbxH*) of the eight genes coding for homeobox-type proteins were not studied in detail in this study, HbxD also appears to affect fungal development. The absence of *hbxD* caused decreased conidial production **(**Fig. 2**)**. Previous genetic analyses have demonstrated that the overexpression of *hbxD* suppresses fungal growth and asexual development, suggesting that HbxD is required for the proper development^50^. The orthologs of HbxD have been studied in other fungi, such as *M. oryzae* and *C. albicans*. The deletion of *GRF10* suppresses hyphal growth and biofilm formation in *C. albicans*^43^. In *M. oryzae*, the absence of *MoHOX7* abolishes the appressorium formation and pathogenicity^40^. These results suggest that HbxD plays diverse roles in filamentous fungi.

In summary, we characterized the roles of genes coding for homeobox-type proteins in the model fungus *A. nidulans*. Our results indicate that HbxA and HbxB have multifunctional roles in governing asexual and sexual developmental cycles and ST production in *A. nidulans*. Additional studies will be needed to provide insight into the genetic regulatory networks and action mechanisms of these transcription factors.

## Methods

### The distribution, phylogenetic analyses, and sequence logos of the Homeobox domains in *Aspergillus*

Three representative *Aspergillus* species, *A. flavus* NRRL 3357, *A. fumigatus* Af293, and *A. nidulans* FGSC A4, were used in this study, and their genomic data were downloaded from AspGD (http://www.aspergillusgenome.org/)^51^. A phylogenetic tree of the homeobox orthologs in these three *Aspergillus* species was generated with MEGA 7 software (Maximum Likelihood method based on the JTT matrix-based model) (http://www.megasoftware.net/). The bootstrap consensus tree inferred from 1000 replicates was assumed to represent the evolutionary history of the taxa analyzed.

### Strains, media, and culture conditions

Fungal strains used in this study are listed in Table S1. For routine procedures, *A. nidulans* was grown using solid or liquid minimal media (MM) with 1% glucose (MMG) and appropriate supplements, such as uridine, uracil, or pyridoxine. Sexual medium (20 g/l glucose, 1,5 g/l glycine, 0.52 g/l MgSO_4_ 7H_2_O, 0.52 g/l KCl, 1.52 g/l KH_2_PO_4_, and 1 ml/l of 1000 x trace element solution; pH 6.5; simplified as SM)^52^ was used to induce the sexual developmental cycle. For ST production, fungal strains were incubated in liquid complete medium (CM) at 30 °C for 7 days. To examine the effects of *hbxA* overexpression, solid MMG (non-inducing), MM with 100 mM threonine (MMT, inducing), or YLC (0.1% yeast extract, 1.5% lactose, 30 mM cyclopentanone) media were used^53^. *Escherichia coli* DH5α cells were grown in Luria-Bertani medium with ampicillin (100 μg/ml) for plasmid amplification.

For routine procedures, wild type (WT) or mutant strains were point-inoculated onto solid MMG plates, and the plates were incubated at 37 °C for 5–7 days in the light or dark as indicated. The photographs of the colonies were taken with a Pentax MX-1 digital camera. To analyze conidiophore structures, fresh conidia were spread onto solid MM plates and incubated at 37 °C for 2 days. The agar containing conidiophores of fungal strains was cut into small blocks and examined under a Zeiss Lab.A1 microscope equipped with AxioCam 105 C and AxioVision (Rel. 4.9) digital imaging software. To count of the number of conidia, conidia were collected from each plate, washed using ddH_2_O, passed through Miracloth (Calbiochem, San Diego, CA) to collect pure conidia, and counted using a hemocytometer. Experiments were performed in triplicate for each strain.

### *hbx* deletion mutant strains

The oligonucleotides used to construct the deletion mutants are listed in Table S2. To generate the deletion mutant strains, gene disruption cassettes were generated by using the double-joint PCR (DJ-PCR) strategies, as previously described^54^. Briefly, the 5ʹ and 3ʹ regions of genes coding for homeobox-type proteins were amplified with primer pairs DF/TR and DR/TF, respectively, from *A. nidulans* FGCS4 genomic DNA. The auxotrophic selection marker *pyrG* (*AfupyrG*) was amplified with primers OHS089/OHS090 by using *A. fumigatus* AF293 genomic DNA as the template. In the overlap PCR, genes coding for homeobox-type proteins disruption cassettes were amplified from the combined 5ʹ and 3ʹ regions of genes coding for homeobox-type proteins and the *AfupyrG* marker by using primer pair NF/NR. For transformation, RJMP 1.59 conidia (1 × 10^8^) were inoculated in liquid YG (MM with 0.5% yeast extract) medium and cultured for 14 h at 30 °C. Afterward, the hyphae were harvested, washed, and incubated with the Vinoflow FCE lysing enzyme (Novozymes) to generate protoplasts^55^. The deletion cassettes were introduced into the protoplasts, and the transformed cells were cultured in the selection medium (MMG without uridine or uracil). The *hbx* genes deletion mutant strains were confirmed by PCR followed by restriction enzyme digestion. At least three colonies per deletion mutation were isolated and phenotypically characterized.

### *hbxA*– or *hbxB*–complemented strains

For the *hbxA*– or *hbxB*–complemented strains, the predicted promoters of *hbxA* and *hbxB* were amplified with the primer pairs OHS0657/OHS0658 and OHS0910/OHS0911, respectively, digested with *Not*I, and cloned into pHS13^56^. The resulting plasmids pYE4.1 and pSH1.1 were introduced into the recipient *ΔhbxA* (TYE14.1) and *ΔhbxB* (TSH1.1) strains to give rise to TYE27 and TSH7, respectively. The complemented strains were verified by PCR and quantitative reverse-transcription (qRT) PCR.

### *hbxA and hbxB* overexpression strains

To generate the *alcA*(p)::*hbxA* and *alcA*(p)::*hbxB* fusion construct, the *hbxA* and *hbxB* open reading frame derived from *A. nidulans* FGCS4 genomic DNA was amplified using the primer pair OHS0743/OHS0744 and OHS1130/OHS1131, respectively, digested with *Bam*HI, and cloned into pHS3, which contains *A. nidulans alcA* promoter^56^. The resulting plasmid pYE5.1 and pSH3.1 were then introduced into TNJ36^57^ to give rise to TYE19 and TSH13, respectively. Strains that overexpress *hbxA* and *hbxB* were selected from the transformants, screened by PCR and qRT-PCR after the induction of the promoter.

### qRT-PCR analysis

For qRT-PCR analysis, samples were collected as previously described^58^. For vegetative samples, WT and mutant conidia were inoculated into liquid MMG and incubated at 37 °C for 12 or 16 h. The mycelia were collected, washed, squeeze-dried, and stored at −80 °C until RNA extraction. For conidium samples, WT and mutant conidia were inoculated onto solid MMG plates and incubated for 48 h. Then, conidia were collected from plates using Miracloth (Calbiochem, San Diego, CA) and stored at −80 °C until RNA extraction. To induce asexual development, WT and mutant conidia were inoculated in liquid MMG and incubated at 37 °C for 16 h. The mycelia were filtered, washed and spread onto solid MMG plate to exposure them to air. The plates were incubated at 37 °C with air-exposure to induce asexual development. Samples were collected at the designated time points following the induction of asexual development. All the samples were collected, squeeze-dried, and stored at −80 °C until RNA extraction.

RNA isolation was carried out as previously described^24,58^. Briefly, each sample was homogenized in TRIzol reagent (Invitrogen Waltham, MA, USA) by using a Mini-Bead beater (BioSpec Products Inc., Bartlesville, OK, USA) and Zirconia/Silica beads (RPI, Mt. Prospect, IL, USA), and then the samples were centrifuged. The supernatants were mixed with cold isopropanol and re-centrifuged. The RNA pellets were washed with 70% ethanol and dissolved in RNase-free dH_2_O. To synthesize cDNA, GoScript Reverse transcriptase (Promega, Madison, WI, USA) was used according to the manufacturer’s instructions. Quantitative PCR was performed using iTaq Universal SYBR Green Supermix (Bio-Rad, Hercules, CA, USA) and CFX96 Touch Real-Time PCR (Bio-Rad). To calculate the expression levels of target genes, the 2^-ΔΔCT^ method was used. β-Actin gene was used as the endogenous control. All the experiments were carried out in triplicate. Primers used for qRT-PCR are listed in Table S2.

### Cleistothecium production assay

To assess cleistothecium production, fungal strains were point-inoculated onto MM or SM agar plates and incubated at 30 °C for 7 days in the dark. The plates were then washed with 70% ethanol to facilitate cleistothecium counting. After washing, ten cleistothecia from each strain were selected, and their diameters were measured using a Zeiss Lab.A1 microscope equipped with AxioCam 105 C and AxioVision (Rel. 4.9) digital imaging software.

### Conidial trehalose analysis

The conidial trehalose assay was performed as previously described^23^. WT or mutant strains were inoculated onto MMG and incubated at 37 °C for 2 days. After incubation, conidia (2 × 10^8^) were collected, washed with ddH_2_O, resuspended in 200 mL of ddH_2_O, and incubated at 95 °C for 20 min. The supernatant was collected by centrifugation, mixed with 0.2 M sodium citrate (pH5.5), and incubated with or without trehalase (3 mU, Sigma), which hydrolyzes trehalose to glucose. The amount of glucose produced from trehalose was assayed with a glucose assay kit (Sigma). Samples untreated with trehalase served as negative controls.

### Stress tolerance assay

The thermal tolerance test was carried out as described previously^26^. Approximately 10^3^ conidia from plates that had been cultured for two-days were placed into ddH_2_O and incubated at 55 °C for 15 or 30 min. After incubation, the conidial samples were diluted, and approximately 100 conidia were spread onto solid MM plates. The plates were incubated at 37 °C for 48 h, and the colonies were counted. The survival rates were calculated as the ratio of the numbers of colonies on the heat-treated and untreated plates. All the experiments were carried out in triplicate.

The oxidative tolerance assay was conducted as described previously ^26^. Approximately 10^3^ conidia were incubated with varying concentrations (0, 0.05, or 0.1 M) H_2_O_2_ for 30 min at 25 °C om temperature. After incubation, each conidial suspension was diluted, and the diluted solution was spread onto solid MM plates and cultured at 37 °C for 48 h. The numbers of colonies were determined and their ratios to the colony number in the untreated control were estimated.

### ST extraction and thin-layer chromatography (TLC) analysis

To extract ST, approximately 10^5^ conidia were inoculated into 5 mL liquid complete medium (CM) and cultured at 30 °C for 7 days in the dark. After cultivation, 5 mL CHCl_3_ was added per sample and the samples were vigorously mixed for 1 min. The organic phases were separated by centrifugation and transferred to new glass vials. Each sample was evaporated, resuspended in 100 μl of CHCl_3_, and spotted onto a TLC silica plate (Kiesel gel 60, 0.25 mm; Merck). The TLC plate was placed into a chamber that contained the toluene:ethyl acetate:acetic acid (8:1:1 v/v) solution to resolve the samples. Afterward, the TLC plate was treated with 1% aluminum hydroxide hydrate (Sigma, St Louis, MO, USA). The images of the TLC plates were captured under UV light (366 nm). The intensities of the ST spots were quantitated using Image J software. Experiments were performed in triplicate for each strain.

### Statistical analysis

Statistical differences between WT (or control) and mutant strains were evaluated using Student’s unpaired *t*-test. Data are reported as mean ± standard deviation (SD). *P* values < 0.05 were considered significant.

## Supplementary information

Supplementary Information.
