# Supplementary material for: Homeobox proteins are essential for fungal differentiation and secondary metabolism in Aspergillus nidulans
Source: Sci Rep. 2020 Apr 8;10:6094. doi: 10.1038/s41598-020-63300-4 (PMC7142095; doi:10.1038/s41598-020-63300-4)
Supplement: Supplementary file 1 — Supplementary Information. [file 41598_2020_63300_MOESM1_ESM.pdf]

Supplementary material

Homeobox genes are essential for fungal differentiation and secondary metabolism in *Aspergillus nidulans*

Sung-Hun Son[1*], Ye-Eun Son[1*], He-Jin Cho[1], Wanping Chen[2], Mi-Kyung Lee[3], Lee Han Kim[4], Dong-Min Han[4], and Hee-Soo Park[1, 5†]

[1] School of Food Science and Biotechnology, Kyungpook National University, Daegu, 41566, Republic of Korea

[2] Department of Molecular Microbiology and Genetics, University of Göttingen, Göttingen, 37077, Germany

[3] Biological Resource Center (BRC), Korea Research Institute of Bioscience and Biotechnology (KRIBB), Jeongeup-si, 34141, Republic of Korea

[4] Division of Biological Sciences, Wonkwang University, Iksan, 54538, Republic of Korea

[5] Department of Integrative Biology, Kyungpook National University, Daegu, 41566, Republic of Korea

[*] These authors contributed equally to this manuscript.

[†] Corresponding Author:
Hee-Soo Park
School of Food Science and Biotechnology, Kyungpook National University
80 Daehak-ro, Buk-gu, Daegu, 41566, Republic of Korea
Tel: +82-53-950-5751;
Fax: +82-53-950-6750
Email: phsoo97@knu.ac.kr

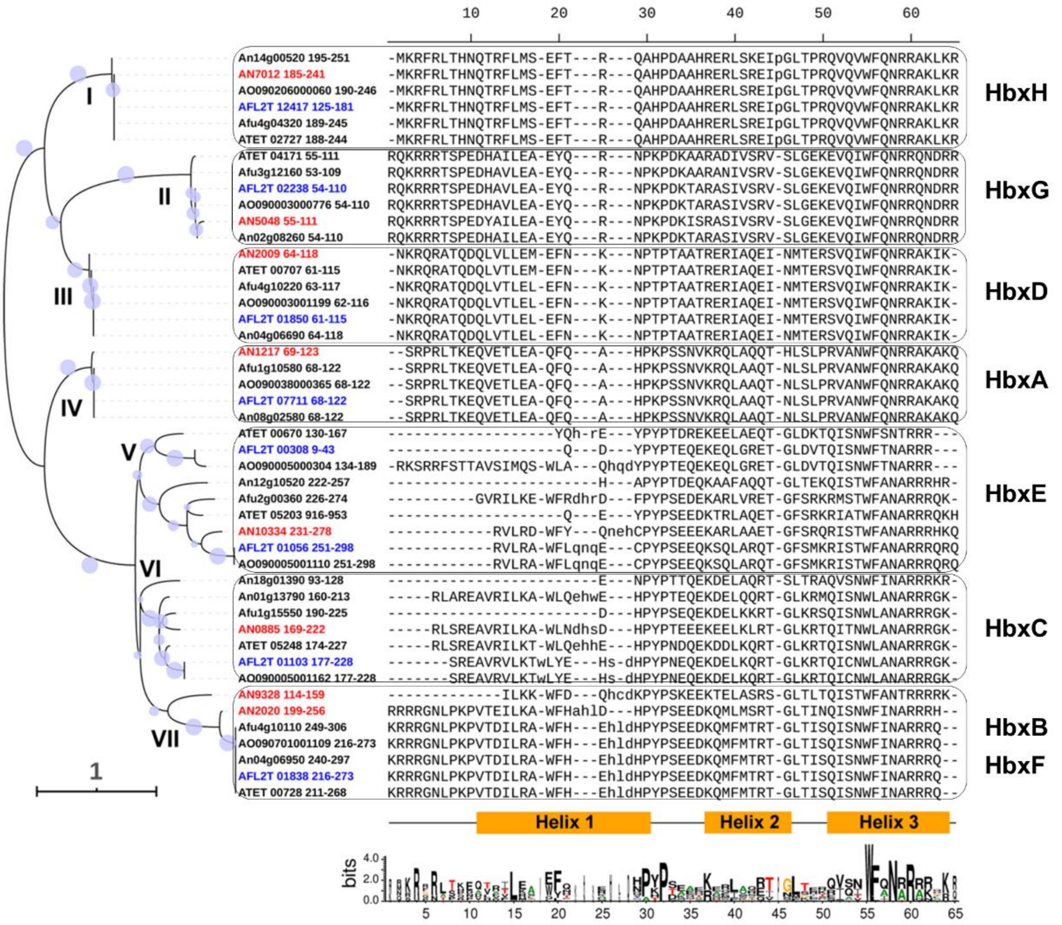

Supplementary Fig. S1: Distribution of Homeobox gene was investigated in six common *Aspergillus* species on genomic level. The genome-wide identification of Homeobox domains was queried from overall coding proteins by using HMMER 3.0 (http://hmmer.org/) with hmmsearch of the profile hidden Markov model derived from the Pfam seed alignment of Homeodomain family PF00046 (https://pfam.xfam.org/family/PF00046). The alignment of all putative Homeodomains were performed by using hmmalign against the profile hidden Markov model and then submitted to IQ-TREE web server for inferring the phylogenetic tree. The best tree selected by the IQ-TREE server was viewed and edited by iTOL (https://itol.embl.de/). The consensus sequence logo of Homeodomains was generated by WebLogo 3 (http://weblogo.threeplusone.com/create.cgi).

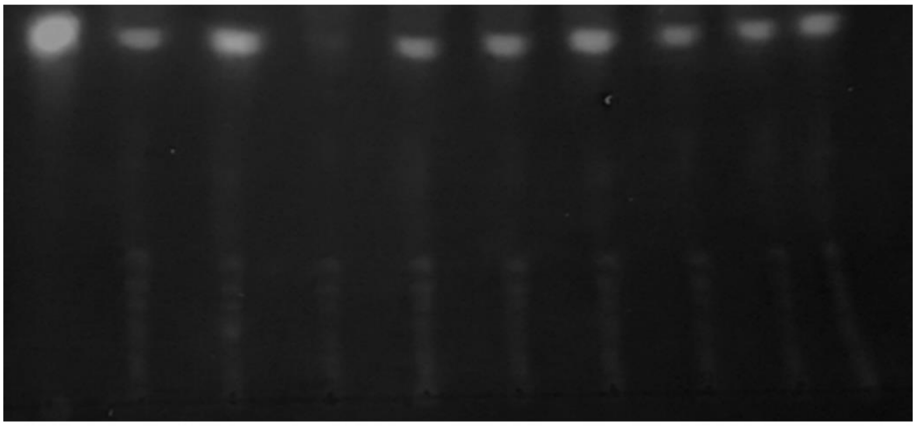

Supplementary Fig. S2. Sterigmatocystin production in the homeobox gene deletion mutant strains in *A. nidulans*. Thin-layer chromatography (TLC) of sterigmatocystin from WT and homeobox gene deletion strains under dark condition for 7 days.

Supplementary Table S1: *Aspergillus* strains used in this study

Strain name	Relevant genotype	References
FGSC4	*A. nidulans* wild type, *veA*$^+$	FGSC[a]
RJMP1.59	*pyrG89*; *pyroA4*; *veA*$^+$	58
TNJ36	*pyrG89; AfupyrG*$^+$; *pyroA4*; *veA*$^+$	52
TYE14.1~3	*pyrG89*; *pyroA4*; Δ*hbxA*::*AfupyrG*$^+$; *veA*$^+$	This study
TSH1.1~3	*pyrG89*; *pyroA4*; Δ*hbxB*::*AfupyrG*$^+$; *veA*$^+$	This study
TSH2.1~3	*pyrG89*; *pyroA4*; Δ*hbxC*::*AfupyrG*$^+$; *veA*$^+$	This study
TYE24.1~3	*pyrG89*; *pyroA4*; Δ*hbxD*::*AfupyrG*$^+$; *veA*$^+$	This study
TSH3.1~3	*pyrG89*; *pyroA4*; Δ*hbxE*::*AfupyrG*$^+$; *veA*$^+$	This study
TYE25.1~3	*pyrG89*; *pyroA4*; Δ*hbxF*::*AfupyrG*$^+$; *veA*$^+$	This study
TSH4.1~3	*pyrG89*; *pyroA4*; Δ*hbxG*::*AfupyrG*$^+$; *veA*$^+$	This study
TSH5.1~3	*pyrG89*; *pyroA4*; Δ*hbxH*::*AfupyrG*$^+$; *veA*$^+$	This study
TYE27.1~2	*pyrG89*; *pyroA*::*hbxA(p)*::*hbxA*::FLAG3x::*pyroA*[b]; Δ*hbxA*::*AfupyrG*$^+$; *veA*$^+$	This study
TSH7.1~2	*pyrG89*; *pyroA*::*hbxB(p)*::*hbxB*::FLAG3x::*pyroA*[b]; Δ*hbxB*::*AfupyrG*$^+$; *veA*$^+$	This study
TYE19.1~3	*pyrG89*; *AfupyrG*$^+$; *alcA(p)*::*hbxA*::FLAG::*pyroA*[b]; *veA*$^+$	This study
TSH13.1~2	*pyrG89*; *AfupyrG*$^+$; *alcA(p)*::*hbxB*::FLAG::*pyroA*[b]; *veA*$^+$	This study

[a] Fungal Genetic Stock Center

[b] The 3/4 *pyroA* marker causes targeted integration at the *pyroA* locus.

Supplementary Table S2: Oligonucleotides used in this study.

Name	Sequence (5′ → 3′)[a]	Purpose
**OHS0089**	GCTGAAGTCATGATACAGGCCAAA	*AfupyrG* Maker_F
**OHS0090**	ATCGTCGGGAGGTATTGTCGTCAC	*AfupyrG* Maker_R
**OHS0643**	CTGCTACTGAGCGCTGGA	*hbxA*_5' DF
**OHS0644**	GCGAAGTAGGGTTGAAGGG	*hbxA*_3' DR
**OHS0645**	*GGCTTTGGCCTGTATCATGACTTCA* AATTGCAGAGCTGAGCCAAG	*hbxA*_Rev with *AfupyrG* TR
**OHS0646**	*TTTGGTGACGACAATACCTCCCGAC* GGGACTCTGTCTCTTATA	*hbxA*_For with *AfupyrG* TF
**OHS0647**	CGATCATCCATCCAATGGCAC	*hbxA*_ 5' NF
**OHS0648**	TGCTGGTGCCTGAGCTAG	*hbxA*_ 3' NR
**OHS0653**	TTGAACCTGTCGACATTCGC	*hbxA*_RT_F
**OHS0654**	GGTCGACAACTCCGTCTTTG	*hbxA*_RT_R
**OHS0665**	TCCCTTGGCCGGTTTCAAC	*hbxB*_5' DF
**OHS0666**	AGCTTGTCCGTCAGTTGGC	*hbxB*_3' DR
**OHS0667**	*GGCTTTGGCCTGTATCATGACTTCA* AGCGAATCGAGACCGAGCA	*hbxB*_Rev with *AfupyrG* TR
**OHS0668**	*TTTGGTGACGACAATACCTCCCGAC* TACACGAGGCAACGTCGAG	*hbxB*_For with *AfupyrG* TF
**OHS0669**	GAAGAAGTTCCCCTCACCC	*hbxB*_ 5' NF
**OHS0670**	CTTCATGACAGTGGCGGTCT	*hbxB*_ 3' NR
**OHS0671**	GGTCAGGTGCGATACCACTA	*hbxB*_RT_F
**OHS0672**	ATGAAACCAGGCCTTGAGGA	*hbxB*_RT_R
**OHS0705**	GTGCCGCTGCCGATTAAA	*hbxC*_5' DF
**OHS0706**	CCCGGTCATACCGGTACAAA	*hbxC*_3' DR
**OHS0707**	*GGCTTTGGCCTGTATCATGACTTCA* TATCTACCACGAGAGCCGC	*hbxC*_Rev with *AfupyrG* TR
**OHS0708**	*TTTGGTGACGACAATACCTCCCGAC* ATGAGGTGGAGTGACGAGC	*hbxC*_For with *AfupyrG* TF
**OHS0709**	TCCTCAAGTACCTGCGCCA	*hbxC*_ 5' NF
**OHS0710**	TGCTGAGGTCTTCGGGAA	*hbxC*_ 3' NR
**OHS0711**	ATGAGCGTCTCAGGATGGAG	*hbxC*_RT_F
**OHS0712**	TTATCGGCACAGGTCTGGTT	*hbxC*_RT_R
**OHS0681**	CAAGACGAATCCCGTCAAGGC	*hbxD*_5' DF
**OHS0682**	CATCCGCGTTACGAATCCG	*hbxD*_3' DR
**OHS0683**	*GGCTTTGGCCTGTATCATGACTTCA* CCAGGATCGGTACTGGCT	*hbxD*_Rev with *AfupyrG* TR
**OHS0684**	*TTTGGTGACGACAATACCTCCCGAC* ACTGACCGGCTAGCATCA	*hbxD*_For with *AfupyrG* TF
**OHS0685**	CTCGTCGTGCACAGAGGTT	*hbxD*_ 5' NF
**OHS0686**	TTGCTGTTGTGGGAGATCGG	*hbxD*_ 3' NR
**OHS0687**	GATGCTTGCGAAGAAGAGCA	*hbxD*_RT_F
**OHS0688**	GTATGCACCATTTGCTCCGT	*hbxD*_RT_R
**OHS0673**	TGCACTCTCTGGCGCAAT	*hbxE*_5' DF
**OHS0674**	AAGCTGGCTGATGGCAGAC	*hbxE*_3' DR
**OHS0675**	*GGCTTTGGCCTGTATCATGACTTCA* GAGATTCGCGCT GTGGAGA	*hbxE*_Rev with *AfupyrG* TR
**OHS0676**	*TTTGGTGACGACAATACCTCCCGAC* TGCATGGTTACG AGCCCGT	*hbxE*_For with *AfupyrG* TF
**OHS0677**	TATCCAGCGCTTGCTGCTTG	*hbxE*_ 5' NF
**OHS0678**	CCGAGGTCCATTTTGACG	*hbxE*_ 3' NR
**OHS0679**	CCAGTGTCGGAGGCTATGAT	*hbxE*_RT_F
**OHS0680**	GATGACCCTTCCAACCCAGA	*hbxE*_RT_R
**OHS0697**	CCAGCACAGCACAGAATACA	*hbxF*_5' DF
**OHS0698**	TGCAGTCGTAGCTGTAGGGA	*hbxF*_3' DR
**OHS0699**	*GGCTTTGGCCTGTATCATGACTTCA* CGACTTCGAAGTGCGAAGCA	*hbxF*_Rev with *AfupyrG* TR
**OHS0700**	*TTTGGTGACGACAATACCTCCCGAC* CACCGCTTG ACGTATCCTGT	*hbxF*_For with *AfupyrG* TF
**OHS0701**	TACCTTGGGCCTCCTGAA	*hbxF*_ 5' NF
**OHS0702**	CGTGAACTTCATGCCGTGT	*hbxF*_ 3' NR
**OHS0703**	CGGGCAACTGTTTACCCAAA	*hbxF*_RT_F
**OHS0704**	TATAGGTTGGCCCGCTTGAA	*hbxF*_RT_R
**OHS0713**	CGCTTGTTCTACCGATACTAGC	*hbxG*_5’ DF
**OHS0714**	CGCTCAGGTAAGACATGCTCA	*hbxG*_3’ DR
**OHS0715**	*GGCTTTGGCCTGTATCATGACTTCA* GCAGCAGAGAGGACAATTAGAG	*hbxG*_Rev with *AfupyrG* TR
**OHS0716**	*TTTGGTGACGACAATACCTCCCGAC* AGCTGCAAGTCAGCAAGC	*hbxG*_Rev with *AfupyrG* TF

**OHS0717**	ACGAGTACAGTCCCTCCTGA	*hbxG_* 5' NF
**OHS0718**	GTCACTCCAGCTCATTCCAGT	*hbxG_* 3' NR
**OHS0719**	GCCACACCTTTCAGTTCACA	*hbxG*_RT_F
**OHS0720**	TCCCTCCTCACACCAAACTC	*hbxG*_RT_R
**OHS0689**	GCTCTGAGTAGTAGCTCCGCTG	*hbxH*_5' DF
**OHS0690**	GCCCCTGCACGCACTTATT	*hbxH*_3' DR
**OHS0691**	*GGCTTTGGCCTGTATCATGACTT* CCTTACCCGCCTAGTCCA	*hbxH*_Rev with *AfupyrG* TR
**OHS0692**	*TTTGGTGACGACAATACCTCCCGAC* GGTGAATGTGGTTGGACACC	*hbxH*_For with *AfupyrG* TF
**OHS0693**	TAT CAG AGG CAT GGC CCT ACT G	*hbxH_* 5' NF
**OHS0694**	TTG CAG GTG CAG TGC AGA GCA	*hbxH_* 3' NR
**OHS0695**	TACCGACGGCCAATGGATTA	*hbxH*_RT_F
**OHS0696**	TTGTGCTTCTTGCTGACCAC	*hbxH*_RT_R
**OHS0910**	*AATT* **GCGGCCGC** GGGTGTGCTGATAGGCAT	*C' hbxB_Not*I_F
**OHS0911**	*AATT* **GCGGCCGC** GGGTGTGCTGATAGGCAT	*C' hbxB_Not*I_R
**OHS0657**	*AATT* **GCGGCCGC** TTCCCGTCAGGCTGTCTG	*C' hbxA_Not*I_F
**OHS0658**	*AATT* **GCGGCCGC** GCCGAAATTGGTGGGGGT	*C' hbxA_Not*I_R
**OHS0743**	*AATT* **GGATCC** ATGAATTATATCCATCATCCATACCCTTTC	*hbxA*_OE_F *with Bam*HI
**OHS0744**	*AATT* **GGATCC** GCCGAAATTGGTGGGGGT	*hbxA*_OE_R *with Bam*HI
**OHS1130**	*AATT* **GGATCC** TGCCACCGGACCCTGACC	*hbxB*_OE_F *with Bam*HI
**OHS1131**	*AATT* **GGATCC** TAGTCTTCGATGAGGTGATGGCTC	*hbxB*_OE_R *with Bam*HI
**OHS0044**	GTAAGGATCTGTACGGCAAC	*Actin*_RT_F
**OHS0045**	AGATCCACATCTGTTGGAAG	*Actin*_RT_R
**OHS0580**	CAAGGCATGCATCAGTACCC	*brlA*_RT_F
**OHS0581**	AGACATCGAACTCGGGACTC	*brlA*_RT_R
**OHS0779**	ATTGACTGGGAAGCGAAGGA	*abaA*_RT_F
**OHS0780**	CTGGGCAGTTGAACGATCTG	*abaA*_RT_R
**OHS0803**	ACAGAGTTGCCTCAGCAGAT	*wetA*_RT_F
**OHS0804**	AGATGTGCCTGTCTGGCTTA	*wetA*_RT_R
**OHS0576**	GGTTGAAGTCGTCGGTTGAG	*tpsA*_RT_F
**OHS0577**	TGGAAACCGATGAGGTCACA	*tpsA*_RT_R
**OHS0048**	CTCAGAGCCGGTCCAGTCAG	*vosA*_RT_F
**OHS0049**	GACTGCAGTACTGGCGGAGT	*vosA*_RT_R

[a] Tail sequences are shown in italics. Restriction enzyme sites are in bold.
